# Differential Impact of CD43 and CD28 on T-Cell Differentiation Depending on the Order of Engagement with the TCR

**DOI:** 10.3390/ijms25063135

**Published:** 2024-03-08

**Authors:** Monserrat Alba Sandoval-Hernández, Nora Alma Fierro, José Ignacio Veytia-Bucheli, Den Alejandro Alvarado-Velázquez, Estefanía Alemán-Navarro, Erika Melchy-Pérez, Constance Auvynet, Iván Imaz-Rosshandler, Jorge Carneiro, Ernesto Perez-Rueda, Yvonne Rosenstein

**Affiliations:** 1 Instituto de Biotecnología, Campus Morelos, Universidad Nacional Autónoma de México (UNAM), Av. Universidad 2001, Col. Chamilpa, Cuernavaca Mor. 62210, Mexico; monserrat.sandoval@ibt.unam.mx (M.A.S.-H.); jose-ignacio.veytiabucheli@unamur.be (J.I.V.-B.); daav_1092@hotmail.com (D.A.A.-V.); estefania.aleman@ibt.unam.mx (E.A.-N.); erika.melchy@ibt.unam.mx (E.M.-P.); constance.auvynet@ibt.unam.mx (C.A.); 2 Posgrado en Ciencias Bioquímicas, Universidad Nacional Autónoma de México (UNAM), Ciudad de México 04510, Mexico; 3 Instituto de Investigaciones Biomédicas, Universidad Nacional Autónoma de México (UNAM), Ciudad de México 04510, Mexico; noraalma@iibiomedicas.unam.mx; 4 Instituto Nacional de Medicina Genómica (INMEGEN), Ciudad de México 14610, Mexico; irosshandler@altoslabs.com; 5 Instituto de Tecnologia Química e Biológica, Universidade Nova de Lisboa, Oeiras, 1169-056 Lisboa, Portugal; jorge.aka.carneiro@gmail.com; 6 Unidad Académica del Estado de Yucatán, Instituto de Investigaciones en Matemáticas Aplicadas y en Sistemas, Universidad Nacional Autónoma de México (UNAM), Mérida 97302, Mexico; ernesto.perez@iimas.unam.mx

**Keywords:** Order of Co-stimulation, CD43, CD28, Transcription Factors, cytokine profiles

## Abstract

The combination of signals from the T-cell receptor (TCR) and co-stimulatory molecules triggers transcriptional programs that lead to proliferation, cytokine secretion, and effector functions. We compared the impact of engaging the TCR with CD28 and/or CD43 at different time points relative to TCR engagement on T-cell function. TCR and CD43 simultaneous engagement resulted in higher CD69 and PD-1 expression levels than in TCR and CD28-stimulated cells, with a cytokine signature of mostly effector, inflammatory, and regulatory cytokines, while TCR and CD28-activated cells secreted all categories of cytokines, including stimulatory cytokines. Furthermore, the timing of CD43 engagement relative to TCR ligation, and to a lesser degree that of CD28, resulted in distinct patterns of expression of cytokines, chemokines, and growth factors. Complete cell activation was observed when CD28 or CD43 were engaged simultaneously with or before the TCR, but ligating the TCR before CD43 or CD28 failed to complete a cell activation program regarding cytokine secretion. As the order in which CD43 or CD28 and the TCR were engaged resulted in different combinations of cytokines that shape distinct T-cell immune programs, we analyzed their upstream sequences to assess whether the combinations of cytokines were associated with different sets of regulatory elements. We found that the order in which the TCR and CD28 or CD43 are engaged predicts the recruitment of specific sets of chromatin remodelers and TFSS, which ultimately regulate T-cell polarization and plasticity. Our data underscore that the combination of co-stimulatory molecules and the time when they are engaged relative to the TCR can change the cell differentiation program.

## 1. Introduction

Immune cells sense environmental cues and alert the organism about potential threats that may alter homeostasis, triggering defense mechanisms that protect the organism against microbes or damaged cells. Among immune cells, T lymphocytes specifically recognize antigens through the antigen-specific receptor (TCR). While the first signal provided by the interaction of the antigenic peptide-MHC complex with the TCR determines the specificity of the response, additional signals delivered by co-receptor molecules that recognize their counter-receptors on antigen-presenting cells are needed for the cells to progress into a differentiation program [1]. The combination of signals from the TCR and the co-stimulatory molecules triggers transcriptional programs that lead to proliferation signals, cytokine secretion, and effector functions. 

One current challenge is to understand the specific contribution and temporality of co-stimulatory molecules in regulating immune responses, as this may lead to the identification of novel targets for stimulating or blocking specific immune responses. CD28, a 44 kDa type I transmembrane glycoprotein belonging to the Ig superfamily, is constitutively expressed on most lymphoid cells. It is a master regulator of T-cell activation and is the most studied co-receptor molecule [2,3,4]. In naïve CD4+ T-cells, the interaction of CD28 with its ligands (CD80/B7.1 and CD86/B7.2) expressed on antigen-presenting cells increases the sensitivity for TCR-dependent signals [5]. As a result, cells progress into the cell cycle and exhibit enhanced cell survival by upregulating the expression of anti-apoptotic proteins [6]. The combination of CD28-mediated signals with those of the TCR results in a transcriptional program that leads to cytokine production and expression of effector molecules that contribute to coordinating the differentiation of distinct subsets of T-cell types [5,7]. CD28-deficient mice exhibit impaired T-cell activation, memory development, and poor T-cell–B-cell crosstalk, underscoring the role of this co-receptor molecule in the establishment of a balanced immune response [8]. Although CD28^−/−^ mice exhibit a defective T-cell activation, when challenged in vivo, they have been shown to develop an effective cytotoxic T-cell response to viral infections with the lymphocytic choriomeningitis virus, suggesting that other cell surface molecules can provide compensatory co-stimulatory signals [9]. One such molecule was found to be CD43 [8]. 

CD43 is a highly abundant sialoglycoprotein expressed by all leukocytes. Its rigid rod-like shape extending 45 nm from the cell surface [10] suggests that it may be involved in the initial contact between cells through its ligands ICAM-1, Siglec-1 and -7, MHC-I, and E-selectin [11,12,13,14,15,16]. Due to its high sialic acid content, it is considered an anti-adhesive molecule, but when interacting with one of its ligands, it functions as a co-receptor molecule that generates intracellular signals that regulate leukocyte activation and migration [17,18]. CD43 alone has been shown to induce gene expression in the absence of additional signals provided by other molecules [19]. In T lymphocytes, the CD43 signaling pathway induces the antigen-independent secretion of IL-2 and pro-inflammatory chemokines and cytokines by activating transcription factors, such as AP-1, NFAT, and NFkB [19,20,21,22]. We reported that when CD43 engagement occurs before that of the TCR, the CD43-mediated signals overcome the interaction between SHP-1 and Lck, and the inhibitory effects of c-Cbl and Cbl-b on TCR signaling [23,24]. As a result, T-cells proliferate, as opposed to cells that were first activated through the TCR, suggesting that the time when the CD43 co-receptor molecule is engaged during T-cell activation modulates T-cell response [23] and underscoring the importance of considering the order of receptor engagement during T-cell activation to understand T-cell behavior.

We aimed to compare the impact of engaging the TCR before, simultaneously, or after CD28 and/or CD43 at different times relative to the TCR engagement on T-cell activation. The timing of CD28, but mostly CD43, engagement relative to TCR ligation resulted in distinct patterns of expression of cytokines, chemokines, and growth factors, as well as of activation-induced markers. Complete cell activation was observed when CD28 or CD43 was engaged before or simultaneously with the TCR, but ligating the TCR before CD43 or CD28 failed to initiate a cell activation program, leading to cytokine secretion. Since the order in which CD43 or CD28 and the TCR were engaged resulted in different combinations of cytokines, we evaluated whether these combinations were predicted to associate with different sets of regulatory elements. Collectively, our data highlight that the combination of co-stimulatory molecules and their engagement timing relative to the TCR can change the cell differentiation program.

## 2. Results

### 2.1. Engaging either CD28 or CD43 with the TCR Results in Robust T-Cell Activation

To better understand the role of CD43 in T-cell activation, we compared the effect of engaging the TCR alone (TCR), simultaneously with CD28 (TCR+CD28), CD43 (TCR+CD43), or with both CD28 and CD43 (TCR+CD28+CD43). We evaluated the expression of CD25, CD40L, CD69, OX40, and PD-1 in primary T lymphocytes isolated from healthy donors at 24 and 72 h post-activation.

**Figure 1:**
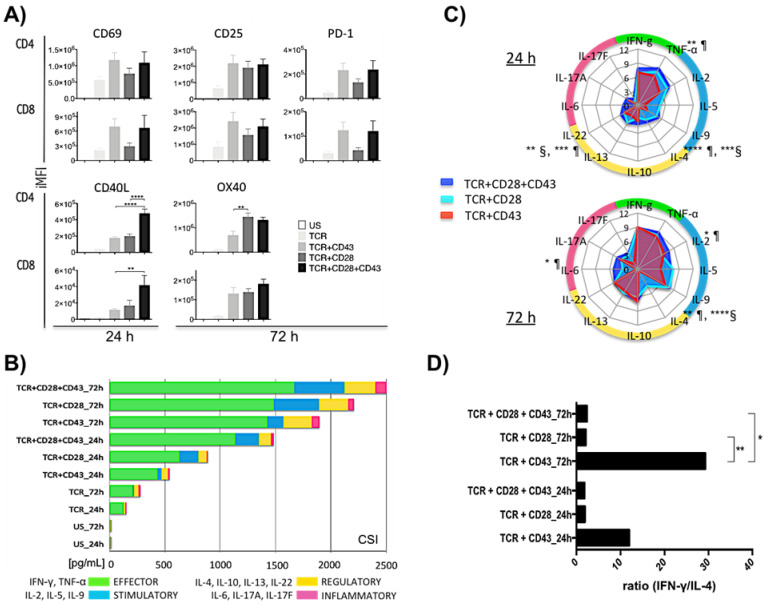
The joint signals of CD28 or CD43, together with the TCR, induce discrete differences in the expression of activation markers and the cytokines secreted. Human T lymphocytes were stimulated as described in the Materials and Methods section. (**A**) The expression of CD69, CD40L, CD25, OX40, and PD-1 was evaluated in the CD4 and CD8 populations at 24 and 72 h iMFI values are shown. (**B**) The cytokines secreted into the supernatant were evaluated at 24 and 72 h. The cytokine strength index (CSI) represents the production (pg/mL) of each category of cytokines by functional category by time and stimulus. Cytokines were classified as: effector (IFN-γ, TNF-α), stimulatory (IL-2, IL-5, IL-9), regulatory (IL-4, IL-10, IL-13, IL-22), and inflammatory (IL-6, IL-17A, IL-17F). (**C**) Cytokine profiles across the three stimuli. The radar graphs show the Log2(ratio) values of each cytokine at each vertex for each stimulus at 24 h and 72 h. Cytokines with statistical significance between co-stimuli are indicated as follows: § TCR+CD43 vs. TCR+CD28; ¶ TCR+CD43 vs. TCR+CD28+CD43. (**D**) IFN-γ/IL-4 ratio at 24h and 72h. Ordinary one-way ANOVA (*p* < 0.05) followed by Tukey’s multiple comparison test was performed for (**A**,**C**,**D**). Significance is marked only between co-stimulus comparisons: * *p* < 0.05, ** *p* < 0.01, *** *p* < 0.001, **** *p* < 0.0001 (*n* = 12 for the TCR+CD28 and TCR+CD43 stimuli, and *n* = 4 for the TCR+CD28+CD43 stimulus).

Cells stimulated only through the TCR, in the absence of co-stimulation, expressed lower levels of all activation markers. As expected for TCR+CD28 stimulated cells, CD69 expression peaked at 24 h after activation (Figure 1A) and was downregulated 72 h after activation (Figures S1 and S2). CD25, OX40, and PD-1 expression reached maximum expression at 72 h, and that of CD40L remained elevated all through. Cells stimulated with the TCR+CD43 stimulus followed the same CD69 and CD25 expression kinetics. Although not significantly different, the expression level of CD69 and PD-1 was greater in response to TCR+CD43 engagement, whereas that of OX40 was significantly greater in response to the TCR+CD28 stimulus; moreover, CD40L expression was significantly greater in response to the triple stimulus (TCR+CD28+CD43) as compared to the TCR+CD28 or TCR+CD43 stimuli, suggesting a synergy between the CD43- and CD28-dependent signaling pathways. CD25 expression peaked in response to all three stimuli (Figure 1A and Figure S2). These data suggest that CD28 and CD43 each contribute specific intracellular signals that differentially regulate the expression of activation markers in CD4+ as well as CD8+ lymphocytes, with the expression of CD69 and PD-1 depending more on CD43-mediated signaling, whereas that of OX40 relies more on CD28-mediated signaling. 

Consistent with their capacity to regulate the expression of activation-induced markers, cells activated with TCR+CD28, TCR+CD43, or TCR+CD28+CD43 released different combinations of an array of cytokines, reaching maximum levels at the 72 h time point. The three stimuli efficiently induced effector cytokines with early and sustained production of IFN-γ. The TCR+CD28+CD43 stimulus significantly produced more TNF-α than the TCR+CD43 stimulus at 24 h, but at 72 h, comparable levels of TNF-α were detected with all three stimuli. In terms of stimulatory cytokines (IL-2, IL-5, and IL-9), the TCR+CD28 and the TCR+CD28+CD43 stimulated cells produced significantly more IL-2 than those stimulated with TCR+CD43 at 24 h, with the TCR+CD28+CD43 stimulus inducing a sustained production and significantly elevated levels of IL-2 from the 24 h time-point. The TCR+CD43 stimulated cells reached comparable levels of IL-2 to that of the TCR+CD28 stimulated cells at 72 h. The levels of IL-5 were lower in response to the TCR+CD43 stimulus both at 24 and 72 h compared to the TCR+CD28 and TCR+CD28+CD43 stimuli, while the levels of IL-9 were lower at 24 h but reached levels comparable to TCR+CD28 and TCR+CD28+CD43 stimuli at 72 h. Regarding the regulatory cytokines, the CD43 co-stimulus did not induce the expression of IL-4, unlike the CD28 co-stimuli and the triple stimulus that induced significantly higher levels of IL-4 within 24 h. The other regulatory cytokines (IL-10, IL-13, and IL-22) were induced mainly in response to TCR+CD28 and TCR+CD28+CD43, with maximum values observed at 72 h after stimulation. Regarding inflammatory cytokines (IL-6, IL-17A, and IL-17F), unlike the TCR+CD28 stimulus that produced more IL-6 at 72 h, the triple stimulus (TCR+CD28+CD43) shows a preferential and sustained induction of IL-6 from 24 h; however, the TCR+CD43 stimulus did not induce IL-6. IL-17A was produced within 24 h in response to the TCR+CD43 and TCR+CD28+CD43 stimuli, only reaching comparable levels at 72 h in response to the TCR+CD28 stimulus. As for IL-17F, it is detected after 24 h in response to TCR+CD28 and TCR+CD28+CD43 but not in response to the TCR+CD43 stimulus. In summary, the TCR+CD43-activated cells secreted less stimulatory cytokines but comparable amounts of regulatory and inflammatory cytokines than the TCR+CD28-activated cells, while the TCR+CD28+CD43 stimulus was faster, more potent, and more balanced than either the TCR+CD43 or TCR+CD28 stimuli *(*Figure 1B,C). Compared with the TCR+ CD28 stimulation, the TCR+CD43 stimulus directed the response towards a Th1 response, as evidenced by the IFN-γ /IL-4 ratio (*p* < 0.01) (Figure 1D). In accordance with the low levels of expression of the activation markers, T lymphocytes produced only small amounts of cytokines, mostly effector ones, when activated through the TCR alone (Figure 1B). 

Altogether, this data suggests that the TCR+CD28 and the TCR+CD43 stimuli strongly activate T lymphocytes but orient the cells towards different functional phenotypes. Notably, the TCR+CD43 activation resulted in a higher CD69 and PD-1 expression level compared to the TCR+CD28-stimulated cells and a cytokine signature consisting mainly of effector, regulatory, and inflammatory cytokines and less stimulatory cytokines than did the TCR+CD28-activated cells which secreted large amounts of all categories of cytokines. Consistent with a balanced expression of activation markers, the triple stimulus resulted in a richer and more balanced cytokine environment, highlighting overlapping and distinctive functions for CD43 and CD28. 

### 2.2. Engagement of CD43 or CD28 before or after TCR Activation Leads to Different Outcomes

**Figure 2:**
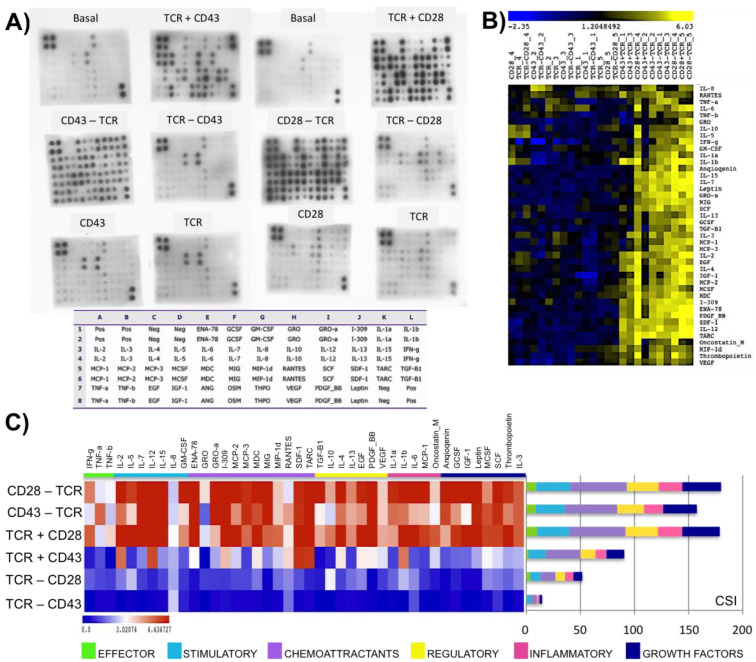
The timing of CD43, CD28, and TCR signaling influences T-cell response. T lymphocytes isolated from the peripheral blood of healthy donors were stimulated with the first stimulus two hours before the second stimulus was applied, after which the cells were incubated for 48 h. (**A**) Cytokines released to the supernatant were detected with a commercial protein array. Data shown are representative of three (CD43 series, upper left) and two (CD28 series, upper right) independent experiments with different donors. The protein distribution is displayed below. (**B**) Unsupervised hierarchical clustering of the semi-quantitative analysis for all donors. Heat map of the log_2_ (ratio) of the relative expression values for each cytokine/protein (rows) through different stimuli (columns). Hierarchical clustering conditions: average linkage algorithm, Pearson correlation metric. (**C**) Effect of temporality on cytokine index. Heat map comparing the categories of cytokines and the cytokine strength index for each stimulus (*n* = 5).

We previously reported that the order in which CD43 and the TCR are engaged results in different signaling responses and proliferation outcomes [23], prompting us to evaluate the production of a set of 42 proteins comprising cytokines, chemokines, and growth factors (collectively referred to as cytokines) in response to CD28 or CD43 ligation before, after, or, simultaneous to TCR engagement. Consistent with data shown in Figure 1, when CD43 was crosslinked simultaneously with the TCR (TCR+CD43), the cells produced a lower quantity of cytokines and a more limited combination of them (about 50%) than the TCR+CD28 stimulated cells after 48 h of incubation. Remarkably, cells produced even more cytokines when CD43 or CD28 was engaged two hours before TCR ligation (CD43-TCR or CD28-TCR). However, engaging the TCR before CD43 (TCR-CD43) or CD28 (TCR-CD28) resulted in the release of only GRO, RANTES, and IL-8. Ligating the TCR, CD43, or CD28 alone did not yield a significant response either. Non-stimulated cells (basal) did not release cytokines, chemokines, or growth factors. The basal levels and the intensity of the response to the different stimuli varied among different donors (Figure 2A and Figure S3).

The unsupervised hierarchical clustering of the data revealed two distinct groups. The first group consisted of cells activated through the TCR, CD43, or CD28 alone and cells where the TCR was ligated for two hours before CD43 (TCR-CD43) or CD28 (TCR-CD28) engagement. This group produced a reduced combination of chemoattractant, effector, and stimulatory cytokines (GRO, RANTES, TNF-β, and IL-8) in low levels. The second group included cells activated by engaging CD28 or CD43 and the TCR simultaneously (TCR+CD43; TCR+CD28) and cells activated by engaging CD43 or CD28 two hours before the TCR (CD43-TCR; CD28-TCR). These stimuli resulted in robust responses, with all six categories (effector, stimulatory, chemoattractants, regulatory, inflammatory, and growth factors) of cytokines abundantly represented. The release of cytokines was highest when CD28 was engaged at the same time or before the TCR. Stimulating CD43 at the same time or before the TCR also produced a strong response, albeit different in quantity and quality compared to CD28 stimulation (Figure 2B,C). Taken together, this data suggests that the timing of CD28 and CD43 engagement relative to the TCR activation fine-tunes T-cell activation and leads to different response programs.

### 2.3. The Timing of CD28 or CD43 Engagement Relative to the TCR Defines Distinct Activation Patterns

**Figure 3:**
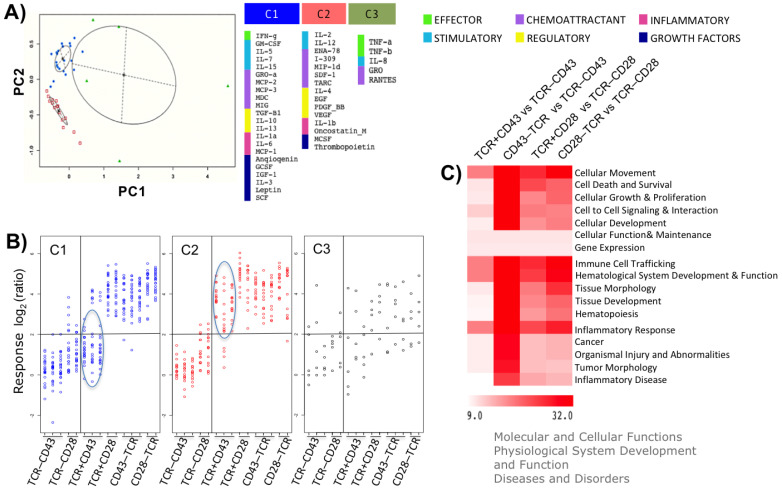
The temporality of the CD43 or CD28 signals relative to that of the TCR defines different activation patterns. (**A**) Clustering and classification of the first two components were achieved using mclust (Supplementary Figure S5B). The best-fitting model was an unconstrained Gaussian mixture model with three components that allowed for variation in ellipsoidal shape, volume, and orientation (VVV). The cytokines were assigned to their respective clusters based on the posterior probabilities. The three clusters were labeled as C1, C2, and C3. Proteins belonging to each cluster are listed. (**B**) Expression profiles. The log_2_ (ratio) protein values belonging to clusters C1, C2, and C3 of each experimental replicate were graphed. Each vertical line of data points represents a different donor (*n* = 5), and each data point is a different cytokine. The color code assigned in A to each cluster was maintained. (**C**) The differential expression (DE) analysis was performed as outlined in the Materials and Methods section through multiple hypothesis testing corrections conducted with Bonferroni (adjusted *p*-values ≤ 0.05), comparing TCR+CD43 with TCR-CD43, CD43-TCR with TCR-CD43, TCR+CD28 with TCR-CD28, and CD28-TCR with TCR-CD28 stimuli. The LogFC (fold change) protein values statistically significant (*p*-values ≤ 0.05) from the differential expression protein analysis were extracted to construct a matrix (Supplementary Table S2), and the enriched diseases and bio functions were retrieved with IPA (Supplementary Table S3). The hierarchical clustering parameters included the average linkage algorithm and the Manhattan correlation metric.

To better understand the different responses we identified, we looked for expression patterns resulting from the different activation protocols, focusing on the (TCR-CD43), (TCR-CD28), (TCR+CD43), (TCR+CD28), (CD43-TCR), and (CD28-TCR) stimuli. After performing a PCA on the correlation matrix of the 42 cytokines, three primary clusters (C1, C2, and C3) were identified (Figure 3A and Figure S5B). Clusters 1 and 2 comprised all categories of cytokines, including effector, stimulatory, chemoattractants, regulatory, inflammatory, and growth factors. However, Cluster 3 only contained effector, stimulatory, and chemoattractant cytokines and lacked regulatory and inflammatory cytokines and growth factors (Figure 3A). The inferred functions for cytokines in Cluster 1 are cell survival, glucose metabolism, cell cycle progression, and proliferation, which ensure a robust T-cell response and memory formation. The cytokines in Cluster 2 are predicted to secure T-cell activation, differentiation, and migration of different leukocyte populations. Similarly, like Clusters 1 and 2, Cluster 3 proteins are predicted to positively regulate leukocyte migration in response to external stimuli.

Figure 3B [(Log2 (ratio) of the three protein clusters across the six stimuli)] shows that, regardless of the order in which TCR, CD43, or CD28 were crosslinked on the cell surface, the proteins of cluster C3 (IL-8, RANTES, GRO, TNF-α, and TNF-β (LTA)) were induced by any of the stimuli. Proteins of Cluster 2 (ENA-78, I-309, IL-12, and the growth factors IL- 2, IL-4, PDGF b, thrombopoietin, VEGF, oncostatin, and EGF) were induced when CD43 or CD28 were engaged before the TCR, with the simultaneous engagement of the TCR and CD43 (TCR+CD43), or the TCR and CD28 (TCR+CD28). On the other hand, proteins of cluster C1 (the growth factors GM-CSF, GCSF, angiogenin, SCF, TGF-β, leptin, IGF1, IL-3, and IL-7, the interleukins IL-6, IL-5, IL-15, IL -1a, IL-13, IL-10, and the chemokines GRO-α, MIG, MCP-1, -2, -3) were induced regardless of the order in which the TCR and CD28 participated (TCR+CD28 and CD28-TCR) but in the case of CD43, only when the CD43 signals preceded those of the TCR (CD43-TCR). Taken together, these results suggest that cluster 3 proteins are secreted regardless of the order in which the molecules participated in the mode of an alarm signal. Meanwhile, the autocrine and paracrine function of the growth factors and interleukins contained in Clusters 1 and 2 complement each other to favor survival and promote a complete activation, proliferation, and differentiation program.

Since engaging CD43 or CD28 at different times relative to TCR ligation resulted in different activation patterns (Figure 2C and Figure S3B), we evaluated whether the combination of cytokines specific to each stimulation protocol would result in distinct biological outcomes. Specifically, we compared the following stimuli: TCR+CD43 and TCR-CD43, CD43-TCR and TCR-CD43, TCR+CD28 and TCR-CD28, and CD28-TCR and TCR-CD28. To identify the functions enriched for the most abundant cytokines of the predominant stimulus in each pairwise comparison, we used the ingenuity pathway analysis (IPA) database. We retrieved the top five “Molecular and Cellular functions”, “Physiological System Development and Function”, and “Diseases and Disorders” (Figure 3C and Table S3). Whether crosslinked simultaneously or before the TCR, CD28, and CD43 led the cells to secrete cytokines in combinations and amounts predicted to elicit all functions. On the other hand, when engaged after the TCR, CD43 was not able to overcome the paucity of response to the TCR signals. Interestingly, although the CD43-TCR stimulus was not the major cytokine producer (Figure 2), the combination and amounts of cytokines released were predicted to induce all the biological functions and diseases better than the CD28-TCR and TCR+CD28 stimuli (Figure 3C).

Collectively, this data suggests that the timing of engaging CD28, but mostly CD43, relative to TCR ligation leads to different cellular outcomes. When CD28 or CD43 are engaged before or simultaneously with the TCR, they provide signals for complete cell activation. However, if the TCR is ligated before CD43 (TCR-CD43) or CD28 (TCR-CD28), cells fail to generate the intracellular signals needed for full cell activation. Different expression patterns of cytokines, chemokines, and growth factors impact cell migration, survival, proliferation, and differentiation, shaping different T-cell immune programs and fine-tuning the immune response.

### 2.4. Different Activation Programs Predict the Usage of Specific Groups of Transcription Regulatory Elements and Different Outcomes

**Figure 4:**
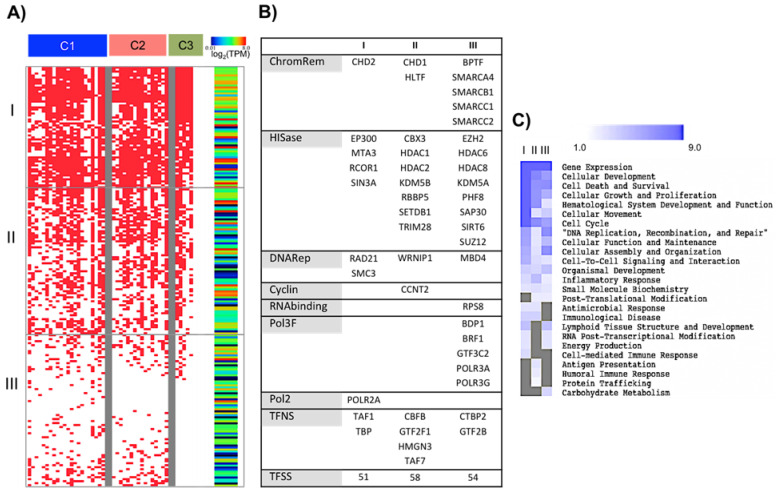
The different activation programs resulting from engaging CD43 or CD28 and the TCR in different order are associated with defined sets of transcriptional regulators. (**A**) *Left panel*: in the matrix of presence and absence of TFs predicted with ENCODE+TRANSFAC, protein-coding genes were ordered based on the profiles defined in C1, C2, and C3. Prediction percentages are ordered from highest to lowest and graphed with a single gradient scale: white 0, red: 1. The frequency of usage of each TF through each profile was calculated to construct a matrix of prediction percentages and a heat map (Figure S6). *Right panel:* The mRNA log_2_ TPM (transcripts per million) values of TFs analyzed in human T lymphocytes by Roadmap complemented the TF’s presence probability and were graphed on a rainbow scale: blue-minor, red-major. (**B**) Molecular functions of the TFs and their diversity between frequency usage groups (I, II, III). Chromatin remodelers, acetyl or methyl histone transferases, DNA repairers, TFNS, and TFSS. (**C**) Diseases and functions associated with each group of TFs (I, II, III) retrieved by IPA (Supplementary Data IPA_D&F_TFs.xlsx). Top “Molecular and Cellular Functions”, “Physiological System Development and Functions”, and “Diseases and Disorders” with *p*-values ≤ 0.05 were graphed as –log (*p*-value) and visualized through a heat map. Rows denote functions, and columns I, II, and III indicate TF prediction percentages. The grey color indicates functions not associated with a TF cluster.

As the order when CD43 or CD28 and the TCR were engaged resulted in different combinations of cytokines, we sought to determine if the co-expression we detected was indicative of co-regulation by specific sets of regulatory elements. To this end, we used the ENCODE and TRANSFAC databases [25,26] to analyze the upstream regions of the 42 protein-coding genes (Supplementary Data Table S4: Neighbour-Promoter_regions, MATCH, Intergenic regions and Total TFs). We created a matrix of presence (1) and absence (0) of transcription factor binding motifs for each gene, disregarding the position and the frequency at which each binding motif appeared for each protein gene and ordering the genes according to the C1, C2, and C3 profiles. We confirmed that the 210 elements we identified had previously been reported as transcribed in human T lymphocytes (Figure 4A, *right panel,* and Supplementary Data Table S4: MatrixTFs & mRNAseqRoadmap, mRNA Roadmap data [27]). Interestingly, the transcription factors predicted to regulate the cytokines also regulate the expression of CD25, CD69, OX40, CD40L, and PD-1 (Supplementary Data Table S4: Matrixmarkers&cytokLegendplex).

The regulatory elements covered all the categories of regulatory elements (chromatin remodelers, histone acetylation/methylation transferases, DNA repairing molecules, general Pol II-associated factor, not site-specific (TFNS), and Pol II transcription factor with sequence-specific DNA binding (TFSS)) [25] and were divided into three main groups (I, II, and III), based on their frequency of usage across the 42 genes of the cytokines array (Figure 4A,B, Figure S6 and Supplementary Data Table S4: MatrixTFsordered percentages). The three groups included different chromatin remodelers, histone acetylation/methylation transferases, DNA repairing molecules, TFNS, and TFSS. Group I contained the essential core to activate the transcription initiation machinery, elements that regulate the long-range chromatin conformation and DNA interactions, and factors associated with hematopoietic and lymphoid cell proliferation and differentiation. Like Group I, Group II included elements regulating the basal transcription machinery, elongation, chromatin remodeling, DNA replication, and cell cycle. This group also contained TFSS associated with metabolism, T-cell activation, and differentiation. Group III contained elements related to Pol III, chromatin organization and stability, transcriptional regulators, and transcription factors that selectively regulate T-cell polarization and metabolism (Figure 4 and Supplementary Data Table S4: dynamic_tables).

Group I elements were predicted to regulate anywhere from 45 to 100% of the genes of the three profiles. Group II usage was predicted for 9–80% of the genes, in comparable proportions for the C1, C2, and C3 profiles. Group III elements were only predicted to be present in 0 to 37% of the genes of the C1 and C2 profiles but not in the C3 profile (Figure 4A, Figure S6 and Supplementary Data Table S4: MatrixTFsordered percentages). Interestingly, the cytokines of the C3 protein profile (TNF-α, TNF-β, IL-8, GRO, and RANTES) were not found to be regulated by Group III regulatory elements (Figure 4A, Figure S6 and Supplementary Data Table S4: MatrixTFsordered percentages), suggesting that these inflammatory genes exhibit accessible promoter regions, histone acetylation, and Pol II occupancy and that only P-TEFb and the release of paused Pol II are needed for activation.

The most enriched gene ontology functions were inferred for each group of regulatory elements. “Gene expression”, “Cell growth and proliferation”, “Survival and death”, “Cellular development and function of the hematopoietic system”, “Cell movement”, and “Cell cycle”, were most significantly enriched for Group I. “DNA replication, recombination, and repair”, “Cell function and maintenance”, Cell assembly and organization", “Structure and development of lymphoid tissue”, “Post-transcriptional modifications of RNA’, and “Energy production” were mainly enriched for Groups I and III. The “Antimicrobial response”, ‘Cell-mediated immune response”, “Antigen presentation”, “Immune disease”, “Humoral immune response”, and “Protein trafficking” functions were concentrated among the regulatory elements of groups I and II (Figure 4C, Supporting Data IPA D&F TFs). The gene ontology functions enriched by the transcription regulatory elements were found to match those enriched for the cytokine profiles.

In summary, this data indicates that the specific time at which the TCR and CD28 or CD43 are engaged affects the activation of signaling pathways that lead to the recruitment of distinct sets of chromatin remodelers and TFSS, which ultimately regulate T-cell polarization and plasticity. Interestingly, a more diverse group of regulatory elements was predicted to be associated with a more complex response.

## 3. Discussion

T-cell activation and functional differentiation require coordinated signals from the T-cell receptor (TCR) and numerous co-receptor molecules to transition to different cell phenotypes. Costimulatory molecules translate information about nutrient availability and the cellular and physiological environment into intracellular signals that modify the T-cell proteomic and transcriptomic landscape, modulating protein synthesis, metabolism, cell cycle progression, and differentiation. As T-cells are increasingly used for immunotherapy, understanding the role played by the molecules participating in T-cell activation and differentiation is a current challenge. We compared the response of human normal peripheral blood T lymphocytes to the TCR and CD28 or CD43 engagement. We considered that the TCR may or may not necessarily encounter its cognate antigen simultaneously and on the same antigen-presenting cell on which co-receptor molecules find their counter-receptors. To mimic these scenarios, we activated the T-cells by ligating the TCR simultaneously with CD28 or CD43 or with CD28 and CD43 at different times, switching the order of the stimuli, and we evaluated the expression of several activation markers as well as cytokine production. 

CD25, CD40L, CD69, PD-1 and OX40 regulate essential processes during T-cell activation, such as cytokine secretion, antibody isotype switching, acquisition of an effector/memory phenotype, regulation of the T-cell migration pattern, cell survival, proliferation, promotion of tolerance, and return to immune homeostasis [28,29,30,31,32]. Engaging the TCR simultaneously with CD28 (TCR+CD28), but also with CD43 (TCR+CD43), or with both CD28 and CD43 (TCR+CD28+CD43) led to robust expression of CD69, CD25, OX40 and CD40L, evidencing the co-stimulatory function of CD43 and CD28, yet with specific differences. When the TCR and CD43 were engaged together, the expression level of OX40 was significantly lower than when the TCR and CD28 were activated together, but that of CD69 and PD-1 was greater. Simultaneously crosslinking CD28, CD43, and the TCR considerably augmented the expression of CD40L, as compared to the TCR+CD28 or TCR+CD43 stimuli, indicating cooperativity between the CD43 and the CD28 signaling. The expression levels of CD69 and PD-1 in response to the triple stimulus suggest that the CD43-mediated signals prevailed over those of CD28, while CD28 controlled the expression of OX40.

Consistent with the expression of activation markers, cells released a variety of cytokines, chemokines, and growth factors in response to TCR, CD43, and CD28 engagement, with the simultaneous engagement of the TCR and CD28 (TCR+CD28) or CD28 and CD43 (TCR+CD28+CD43) resulting in a more robust response overall compared to the TCR+CD43 stimulus. TCR+CD28-activated cells secreted large amounts of cytokines in all categories, resulting in a more balanced response. Unlike CD28 signaling, CD43-dependent signaling induced a polarized Th1-Th17 and IL-10 response. Whether the CD43-induced PD-1 expression is related to this phenotype remains to be investigated. Notably, the TCR+CD43 stimulated cells released large amounts of IFN-γ but no IL-4. Reflecting the high expression of CD40L and OX40, the TCR+CD28+CD43 stimulus yielded a more balanced and robust (approximately 40% greater) response than that induced by the TCR+CD28 stimulus, with a significant proportion of effector and stimulatory cytokines. Consistent with the fact that we stimulated total T-cells, the combination of cell surface markers and secreted cytokines became more complex as the differentiation process progressed, reflecting the variety of T-cell populations present.

We have shown that if the signals from CD43 and TCR are given simultaneously or if CD43 ligation occurs before TCR ligation, the lymphocytes undergo proliferation [23]. Remarkably, engaging CD43 or CD28 two hours before the TCR or simultaneously with the TCR resulted in the robust production of all cytokines. In contrast, if the TCR is engaged before CD43 or CD28, or if the TCR, CD28, or CD43 are engaged individually, a limited cytokine response is observed, further showing that the activation profile of a T-cell can be influenced by the order in which CD43 and CD28 are engaged relative to the TCR. Interestingly, engaging the TCR, CD43, or CD28 alone promoted the release of IL-8, RANTES, GRO, TNF-α, and TNF-β (LTA), known to promote inflammation and leukocyte migration in response to external stimuli, possibly functioning as a warning signal for the organism, but not providing a full activation signaling. These findings highlight the importance of the order in which co-receptor molecules such as CD43 are engaged for T-cell activation.

Consistent with the functions of OX40, CD40L, and CD25, cytokines in the C1 group were inferred to promote cell survival, glucose metabolism, cell cycle progression, and proliferation, ensuring robust activation and memory formation. This group included growth factors and cytokines, such as IL-3, IL-7, and IL-15, which are associated with survival and memory, while the expression of IFN-γ and IL-10 reflects an effector response that has not yet been polarized. Cytokines of the C2 profile participate in T lymphocyte activation and differentiation and the migration of different populations of leukocytes. Like the C1 and C2 profiles, the C3 profile was predicted to promote leukocyte migration in response to external stimuli and to define the activation program of the different T lymphocyte subsets.

Accordingly, the differences detected in response to the different activation protocols reflected the participation of different sets of transcription factors. We identified three groups of transcription factors associated with the transcription of genes of the C1, C2, and C3 profiles. The complexity and magnitude of the response correlated with the diversity of regulatory elements, with C1 having the highest diversity and C3 the lowest. Transcription factors were classified into “top”, “middle”, or “bottom” levels based on the hierarchical organization proposed by the ENCODE project [33]. Although all three hierarchies were present in each group, their proportions varied. In Group I, most transcription factors were in the “top” and “middle” hierarchies; in Group II, the three hierarchies were present in equal proportions, while Group III had more “bottom” hierarchy transcription factors. The three groups consisted of different but complementary sets of chromatin remodelers, histone acetylation/methylation transferases, DNA repair molecules, TFNS, and TFSS, ensuring the transcription of a given gene in response to a given stimulus. Even though this was a predictive analysis in which only the presence or absence of transcription factors was considered, finding a greater variety of transcription factors associated with greater functional response complexity, although intuitive, was surprising. Overall, the inferred biological functions of these transcription factors match those inferred for the cytokines.

Our findings are in line with previous studies showing that CD43 crosslinking alone can trigger gene transcription at levels comparable to those induced by CD28/TCR stimulation [19]. Under our experimental conditions, we found that similar to CD28, the engagement of CD43 together with the TCR led to significant T-cell activation, yet with subtle nuances. CD43 primarily regulated CD69 and PD-1 expression and directed the cells toward a Th1-Th17 response that is balanced or regulated by IL-10 and IL-22. On the other hand, CD28 upregulated OX40 expression, leading to a more balanced cytokine profile, and engaging CD43, CD28, and the TCR simultaneously resulted in a more robust and balanced response. Our data also suggest that the intensity and quality of the response are determined by the time when CD43 was ligated relative to the TCR. This emphasizes that the moment when CD43 is activated plays a critical role in determining the cell response. Additionally, our analysis predicted that the different combinations of cytokines resulting from the different stimuli are associated with different sets of regulatory elements, leading to different activation programs. 

We did not analyze the response of specific T-cell subsets to different stimuli. However, our assessment of the response of unfractionated peripheral blood T-cells from healthy donors shows the complexity of T-cell response and how the engagement of different co-receptor molecules at different times contributes to T-cell activation. Besides its co-receptor signaling function, CD43 also participates in T-cell trafficking. It is plausible to envision that as cells migrate to the lymph nodes or the site of injury, CD43 prepares the cells by interacting with its ligands, complementing the TCR signals and those of other co-receptor molecules, such as CD28, not known to participate in cell trafficking, to direct the cells towards an activation program. Also, due to its elongated and rigid extracellular domain, CD43 may be one of the first molecules of the T-cell membrane to make contact with antigen-presenting cells or target cells, further supporting the notion that not all molecules participate at the same time, and the sequence in which they engage is important for activation and cell fate.

In conclusion, our data show that as the joint signals of CD28 and the TCR, the joint signals of CD43 and the TCR result in robust T-cell activation, although leading to different cellular outcomes. Particularly, the signals from CD43 and TCR negatively regulate Th2 cell differentiation, but when combined with CD28, they result in a faster, sustained, and less polarized response than either the TCR and CD43 or TCR and CD28 signals. We show that the timing of TCR and CD28 or CD43 engagement affects the activation of signaling pathways associated with defined sets of transcriptional regulators, which ultimately regulate cell polarization and plasticity. 

## 4. Materials and Methods

### 4.1. Cell Activation

Platelet-depleted leukocyte concentrates from anonymized healthy donors were obtained from the Centro Estatal de la Transfusión Sanguínea in Cuernavaca (México) and were processed according to institutional guidelines. Mononuclear cells were separated by density centrifugation, and T lymphocytes were further purified by negative selection with a Pan T-Cell Isolation Kit (Miltenyi Biotec GmbH, Berisch Gladbach, Germany) (>95% OKT3^+^). Cells were arrested in 2% FCS-supplemented RPMI overnight. For simultaneous TCR+CD43 co-stimulation experiments, the anti-CD3 (OKT3, American Type Culture Collection, Manassas, VA, USA; anti-CD3, IgG2a) and the anti-CD43 (L10, a murine IgG1 mAb that recognizes human CD43 [34]) were purified from ascites or from Caltag Laboratories, Burlingame, CA, USA. Antibodies were immobilized on the surface of the wells of 48-well plates by incubating the antibodies (10 μg/mL, 100 μL/well in PBS) for 2 h at 37 °C. Wells were washed three times with PBS to remove the unbound antibody before seeding the cells (5 × 10^5^ cells/500 μL RPMI 10% FCS/well). For CD28 co-stimulation, soluble anti-CD28 (CD28.2, 2 μg/mL, Tonbo Biosciences, San Diego, CA, USA) and anti-mouse IgG1 (2 μg/mL, SouthernBiotech, Birmingham, AL, USA) were added to the OKT3-plate-bound wells at the onset of the experiment. For experiments where the order of participation of the TCR and CD28 or CD43 were evaluated, cells were stimulated as previously described [23]. In brief, cells in a 96-well plate (2 × 10^4^ cells/well) were first stimulated with mAbs (4 µg/mL) against the TCR, CD43, or CD28, and class-specific secondary antibodies (4 µg/mL) for two hours. Then, cells were washed to remove excess antibodies, and the second stimulus was applied. Cells submitted to only one stimulus (CD43, CD28, or the TCR individually) were subjected to the same manipulations throughout the experiment. Cells were incubated at 37 °C with 5% CO2 for the indicated times.

### 4.2. Flow Cytometry

Cells were washed with FACS solution (PBS, 0.5% bovine serum albumin, and 0.1% sodium azide) for fluorescent antibody staining. FcRs were blocked with 10% human serum at 4 °C for 30 min. Cells were stained with PE anti-human CD3d (clone 7D6, Caltag Laboratories,), APC/Cy7 anti-human CD4 (clone OKT4, BioLegend, San Diego, CA, USA), Brilliant Violet 510 anti-human CD8 (clone SK1, BioLegend), APC anti-human CD25 (clone BC96, BioLegend), PE anti-human CD69 (clone FN50, BioLegend), FITC anti-human CD40L (clone 24-31, BioLegend), Pacific Blue anti-human PD-1 (clone EH12.2H7, BioLegend), and PerCP/Cy5.5 anti-human OX40 (clone Ber-ACT35, BioLegend). Cells were fixed with 2% paraformaldehyde in PBS and stored at 4 °C until analysis. Samples were acquired on a FACSCanto II (BD Biosciences, San Jose, CA, USA) flow cytometer with the FACSDiva (BD Biosciences, San Jose, CA, USA) software version 6.1.3 and analyzed with FlowJo (version 8.7). Data are shown as so-called integrated mean fluorescence intensity, iMFI, which is calculated as the relative frequency of positive cells for a given marker multiplied by the mean fluorescence intensity of cells positive for that marker. This aggregate quantity is sensitive to changes in the relative frequency and the MFI of positive cells [35]. 

### 4.3. Cytokine Detection

Cytokines and chemokines released into the supernatant were detected by an immune-based multiplexed immunoassay (LEGENDplex™ Human Th Cytokine Panel (13-plex), BioLegend) or with a 42-cytokine array (RayBio^®^ Human Cytokine Antibody Array 3) from RayBiotech, Norcross, GA, USA following the manufacturers’ instructions. A cytokine strength index (CSI) was calculated as the sum of the concentration of the cytokines produced within each functional category. Cytokines were classified as effector (IFN-γ, TNF-α, and TNF-β), stimulatory (IL-2, IL-5, IL-7, IL-8, IL-9, IL-12, IL-15, and GM-CSF), inflammatory (IL-1a, IL-1b, IL-6, IL-17A, IL-17F, MCP-1, and Oncostatin_M), regulatory (IL-4, IL-10, IL-13, IL-22, TGF- β1, EGF, PDGF-BB, and VEGF), chemoattractants (ENA-78, GRO, GRO-α, I-309, MCP-2, MCP-3, MDC, MIG, MIP-1d, RANTES, SDF-1, and TARC) and growth factors (angiogenin, GCSF, IGF-1, leptin, MCSF, SCF, thrombopoietin, and IL-3) [36,37,38].

### 4.4. Image Analysis and Data Normalization

Films of the 42-cytokine antibody arrays were scanned with a Molecular Imager Gel Doc^TM^XR + Imaging System (Bio-Rad, Hercules, CA, USA) and the Image Lab software (version 5.2.1). Densitometric analysis of 8-bit images was performed with the ImageJ software (National Institutes of Health, Bethesda, ML, USA, version 1.48). Raw intensities were normalized across biological replicates using quantiles [39]. Relative expression values were computed as the log_2_ (ratio) of the intensity measured for a given experimental value divided by its corresponding intensity in non-stimulated cells. *i* = 1–44 is the number of proteins, and the array positive or negative controls; *j* = 1–5 refers to the number of arrays, each corresponding to a different individual (Supplementary Table S1). Data distribution before and after normalization and positive and negative control values across samples are included in Figure S4.

### 4.5. Correlations, Principal Component Analysis, and Protein Expression Signatures

The analysis of 42-cytokine expression signatures was focused on data from cells stimulated with TCR-CD43, TCR-CD28, TCR+CD43, TCR+CD28, CD43-TCR, and CD28-TCR. The log_2_ ratios of cytokine intensity values of 15 replicates were used to obtain the Spearman correlation coefficient between the 42 cytokines. This correlation matrix was the input for principal component analysis (PCA) without performing scaling before calculating the PCA. The mclust R package [40,41] was used to perform clustering and classification on the top principal components. The Bayesian information coefficient (BIC) was used to select the Gaussian mixture model that best fits the data (a large BIC score indicates strong evidence for the corresponding model) [40]. The resultant protein clusters were studied as cytokine expression signatures or profiles.

### 4.6. Differential Protein Expression Analysis

The differential expression protein analysis was performed with the Limma Bioconductor package [42,43]. A linear model of log_2_ ratios was fitted, and a moderated *t*-test was implemented. Bonferroni correction was used to control for multiple hypotheses testing [44]. *p*-values ≤ 0.05 were considered to indicate statistical significance (Supplementary Table S2). All computations were done with the R statistical software version 4.2.0.

### 4.7. Transcription Factors Prediction

The upstream sequences were retrieved for each of the 42 protein-encoding genes in the array considering the plus strand, the first exon of each gene, and the boundaries between the start and the end of the neighboring gene (Supplementary Data Table S4: Neighbor-Promoter_Regions). The list of transcription factors (TFs) potentially associated with each gene was obtained from the ENCODE project [25] (https://www.encodeproject.org/) and the TRANSFAC database [45] (http://gene-regulation.com/pub/programs.html#match). Data available from ChIPseq experiments were obtained using *“track qualified Uniform TFBS ChIP-seq Clusters (161 factors*)” with Factorbook Motifs, under group–regulation, Table-wg Encode RegTfbsClusteredV3, linked to the Human Feb. 2009 assembly (GRCh37/hg19) (Supplementary Data Table S4: Nomenclature). Candidate cis-Regulatory Elements (ccREs) in the intergenic regions were identified (https://screen.wenglab.org/) with [27] *Human Feb. 2009 (GRCh37/hg19) and Human Dec. 2013 (GRCh38/hg38)* (Supplementary Data Table S4: Intergenic regions&Total TFs). Additional putative TF binding sites searching the promoters (from 200 bp downstream to 1000 bp upstream of the TSS) were predicted with the positional weight matrix library from TRANSFAC Professional (free trial) (Match^TM^, geneXplain GmbH, Wolfenbüttel, Germany) [45], using the Ensembl, version 70 (equivalent in sequence to the Human Feb. 2009 assembly (GRCh37/hg19) used by ENCODE). The predefined profile (non-redundant vertebrate) with cut-offs by default was used to minimize the sum of the false positive and false negative error rates. Only binding sites with a core match score = 1 and matrix match score = average (throughout all promoters) + 1 SD were considered (Supplementary Data Table S4: MATCH). Predictions were further filtered and curated to exclude nomenclature duplications and match the ENCODE nomenclature (Supplementary Data Table S4: Nomenclature). TFs belonging to the same family of proteins and recognizing the same PWM were merged. The ENCODE and MATCH datasets were combined into a matrix of presence (1) and absence (0) to determine the distribution of these binding sites across the 42 protein-encoding genes, disregarding the position and the number of times each TF binding motif appeared for each protein-encoding gene (Supplementary Data Table S4: MatrixTFs&mRNAseqRoadmap).

### 4.8. Ingenuity Pathway Analysis

The Ingenuity Pathway Analysis (IPA) software (https://analysis.ingenuity.com/pa/installer/select) [46] was used to identify the enriched biological functions from both the proteins (42 cytokine array) that were differentially expressed (DE) between co-stimulus comparisons and from the sets of predicted transcription factors. The LogFC values of proteins from the DE analysis with *p*-values ≤ 0.05 were extracted as a matrix (Table S2). The enriched biological functions for the most abundant cytokines of the predominant stimulus in each pairwise comparison were retrieved through the “Downstream Effects Analysis” algorithm defined by *p*-values ≤ 0.05, and plotted as –log (*p*-value) (Table S3) as a heat map. In the case of the TFs, the matrix of presence and absence was ordered based on the cytokine expression profiles defined by mclust (see Section 4.5). The presences were converted into TFs prediction percentages across the expression profiles. Finally, enriched functions of the TFs throughout the cytokine profiles were retrieved (Supplementary Data Table S4: TFspercentages).

### 4.9. Clustering Analysis

Hierarchical clustering analyses were performed with the MeV_4_8 (MultiExperiment Viewer) version 10.2 application with different metrics, as indicated.

### 4.10. Statistical Analysis

Statistical analysis, including mean values, SEM values, and *p* values, was performed using GraphPad Prism version 6.0c (GraphPad Software). For more than two groups, one-way ANOVA with Tukey post hoc test was performed. *p* values less than 0.05 were considered significantly different (* *p* < 0.05; ** *p* < 0.01; *** *p* < 0.001). The mean (±SEM) is indicated with horizontal lines. n represents the number of biological replicates unless otherwise stated. Statistical details for each experiment can be found in the corresponding figure legends. All error bars represent the SEM.

## Data Availability

Data are contained within the article and Supplementary Materials.

## Supplementary Materials

The following supporting information can be downloaded at https://www.mdpi.com/article/10.3390/ijms25063135/s1.
