# Supplementary material for: Differential Impact of CD43 and CD28 on T-Cell Differentiation Depending on the Order of Engagement with the TCR
Source: Int J Mol Sci. 2024 Mar 8;25(6):3135. doi: 10.3390/ijms25063135 (PMC10970108; doi:10.3390/ijms25063135)
Supplement: Supplementary file 1 [file ijms-25-03135-s001.zip › Supplementary Figures.pdf]

SUPPLEMENTARY FIGURES

Differential Impact of CD43 and CD28 on T-Cell Differentiation Depending on the Order of Engagement with the TCR

Monserrat Alba Sandoval-Hernández et al.

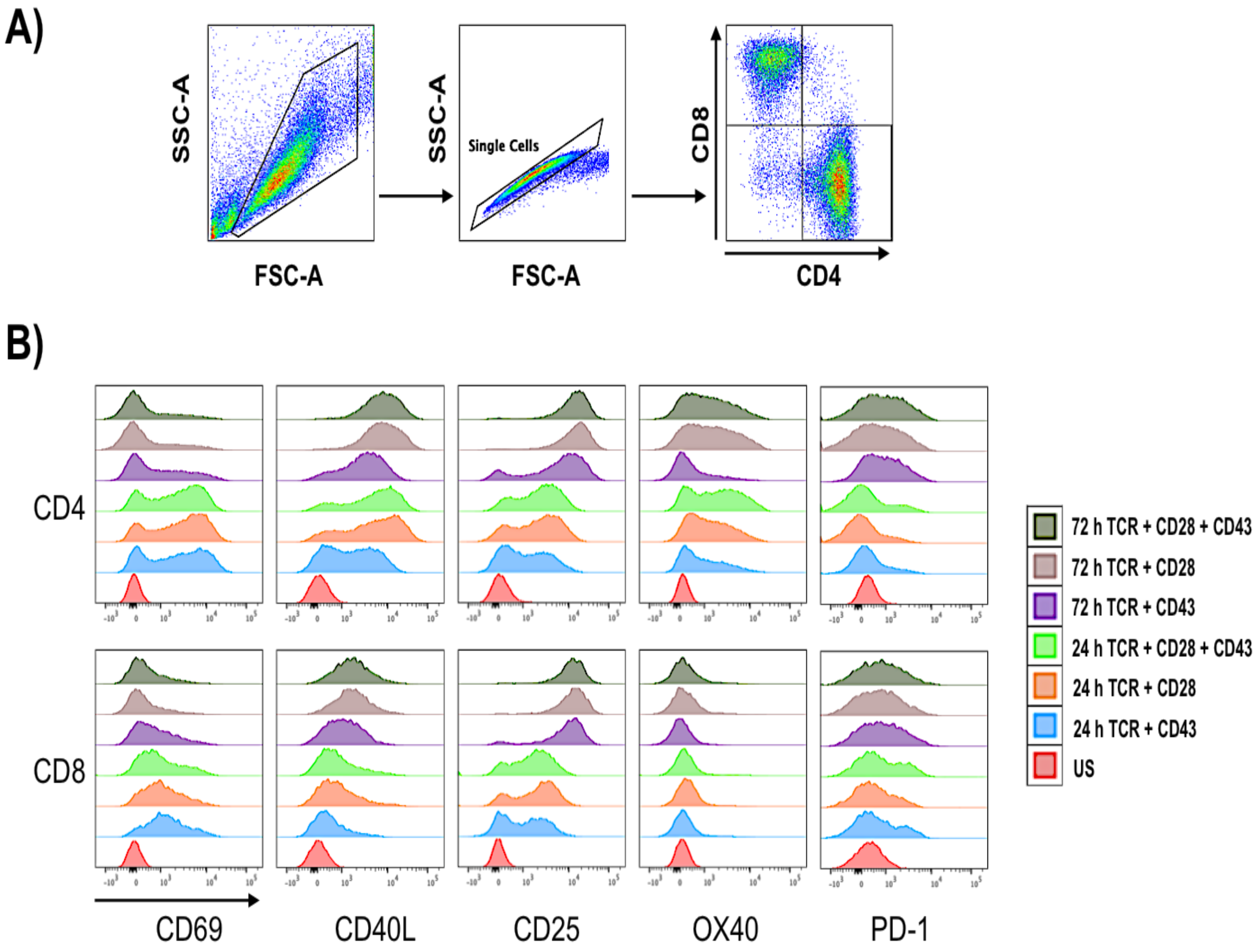

Supplementary Figure S1.
Expression of activation-induced markers in response to TCR+CD28, TCR+CD43, or TCR+CD28+CD43 stimulation. Human T lymphocytes were stimulated as described in the Materials and Methods section. **A)** workflow analysis stages for all donors: data preprocessing, FCS files imported, Compensated sample files were analyzed starting by creating an SSC vs. FSC plot and applying a linear transformation for the scatter channels and selecting $CD4^+$ T and $CD8^+$ lymphocytes gates. **B)** The CD69, CD40L, CD25, OX40, PD-1 expression was assessed in the CD4 and CD8 populations at 24 and 72 h. Mean fluorescence intensity (MFI) values were obtained for each activation marker throughout the evaluated stimuli. Multiple histogram comparisons between stimuli represent a summary of the analysis following the order and color code indicated. Data shown are representative of 1 out of 12 independent donors.

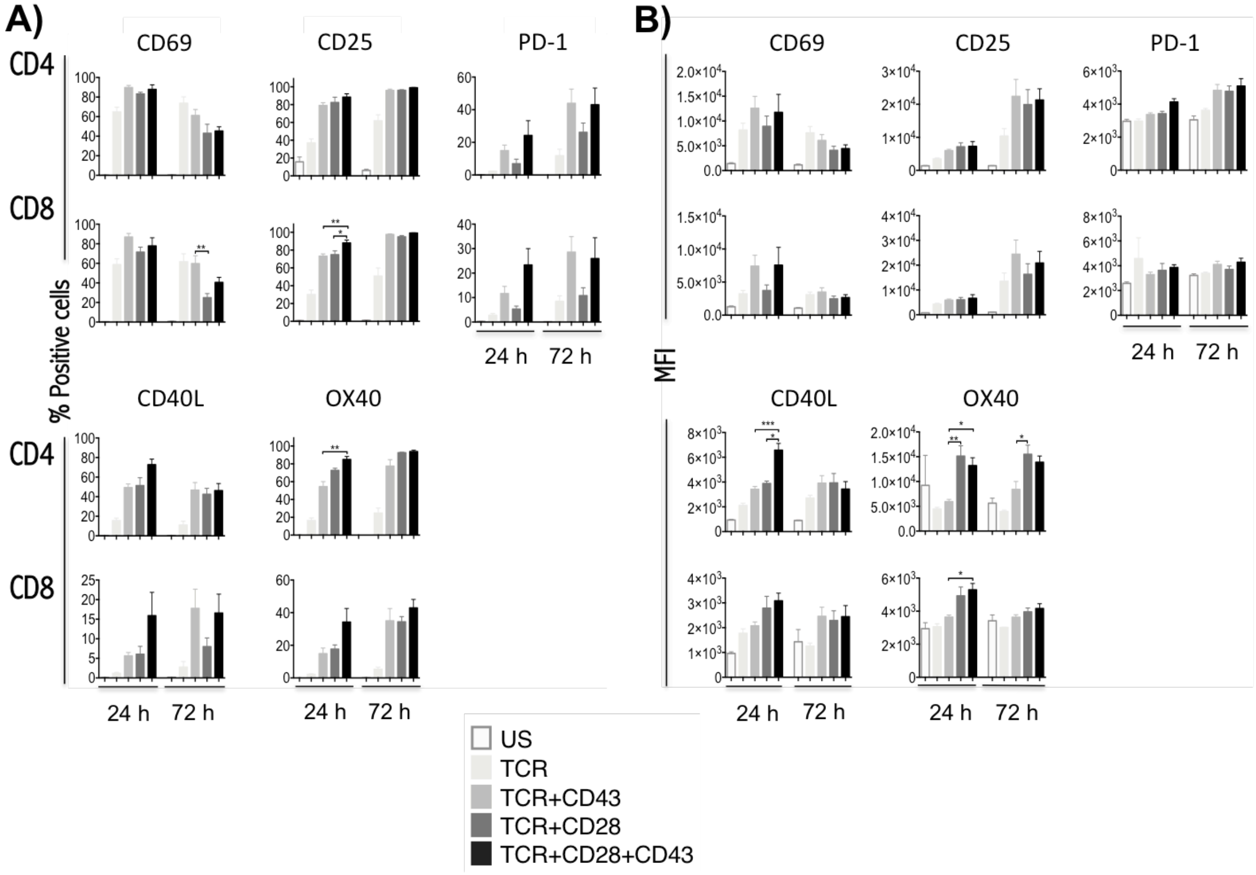

Supplementary Figure S2. Human T lymphocytes were stimulated as described in the Materials and Methods section. Following the workflow analysis described in Supplementary Figure S1, the expression of CD69, CD40L, CD25, OX40, and PD-1, in the CD4 and CD8 cells of all donors at 24 and 72 h post-stimulation and expressed as Percentages (%) of positive cells for each marker **(A)**, and Mean fluorescence intensity (MFI) **(B)**. Significance is marked only between co-stimulus comparisons: *p < 0.05, **p < 0.01, ***p < 0.001, **** p < 0.0001. n=12 for all the TCR+CD28 and TCR+CD43 stimuli, and *n*=4 for the TCR+CD28+CD43 stimulus.

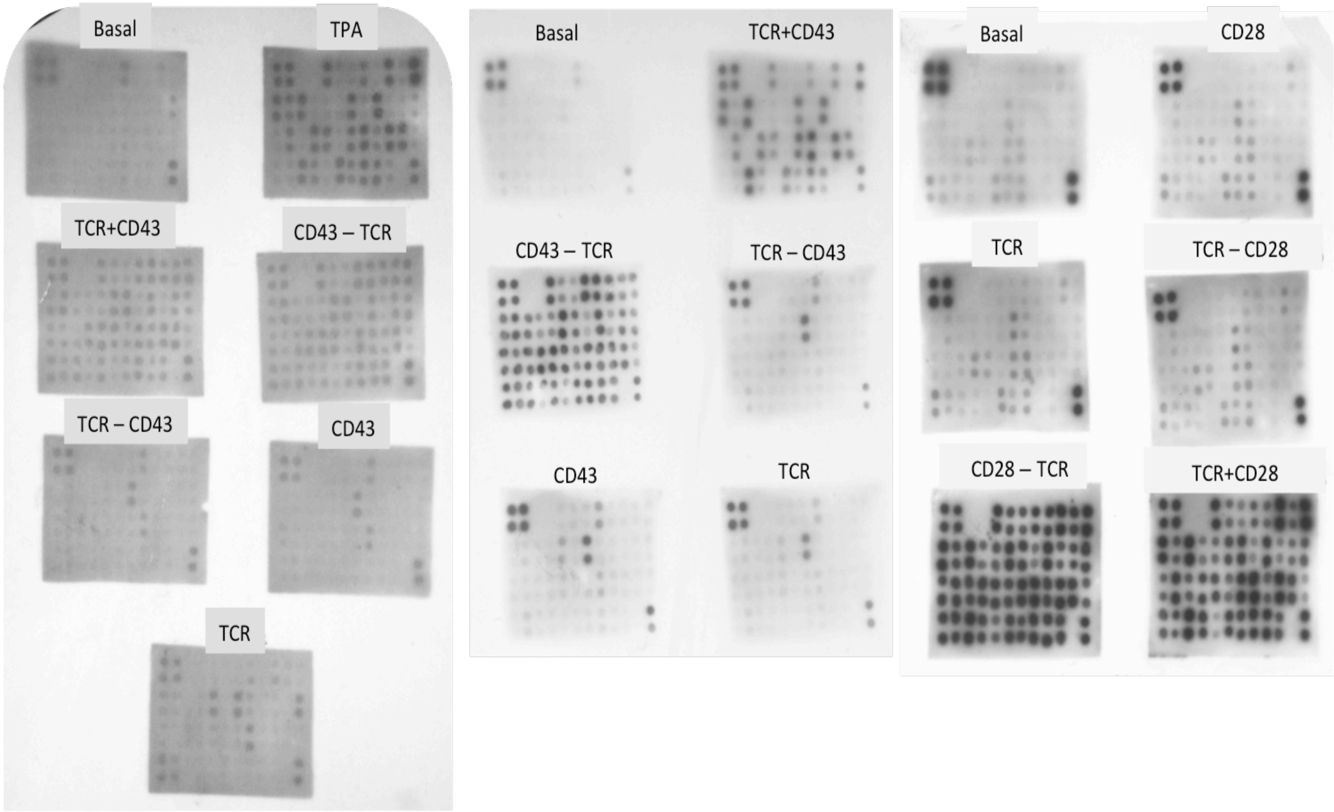

Supplementary Figure S3. The timing of CD43, CD28, and TCR signaling influences the T-cell response.

T lymphocytes isolated from peripheral blood of healthy donors were stimulated with the first stimulus two hours before applying the second stimulus, after which the cells were incubated for 48 hours. Cytokines released into the supernatant were detected with a commercial protein array. Data shown are the two (CD43) and one (CD28) complementary series of Figure 2A, with different donors.

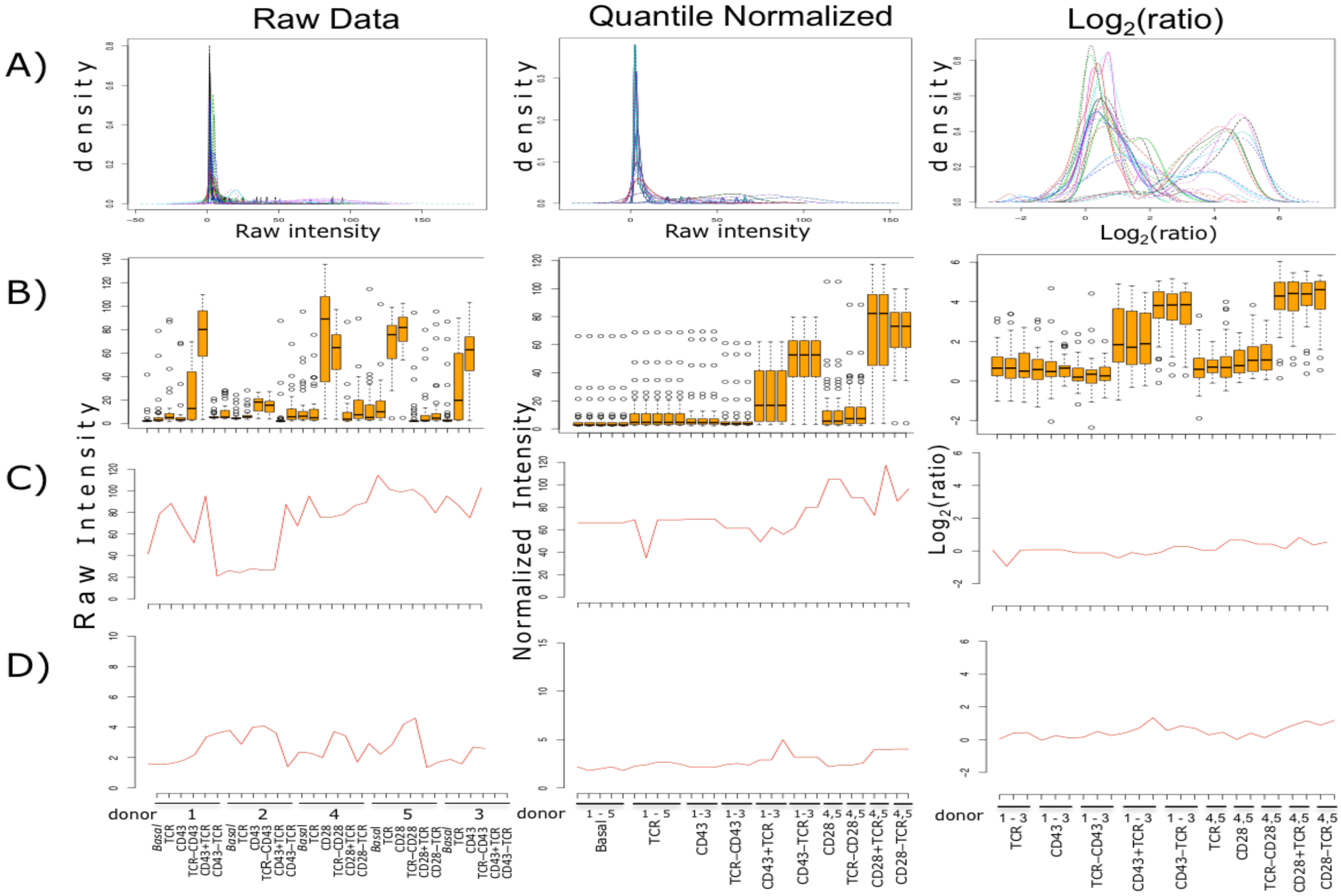

Supplementary Figure S4. Data normalization.

Films of the cytokine antibody arrays of the three (CD43) and two (CD28) independent experiments with different donors from **Figure 2A** and **Supplementary Figure S3** were scanned. The normalization process started with the raw data shown in the left column: Basal, TCR, CD43 [CD43, TCR-CD43, CD43+TCR, CD43-TCR] and CD28 [CD28, TCR-CD28, CD28+TCR, CD28-TCR] stimuli. Raw intensities were normalized across biological replicates using quantiles (center column). Relative expression values were computed as the log2 ratio of a given experimental value divided by its corresponding value in non-stimulated cells. A and B) Data distribution. C) Positive controls across samples. D) Negative controls across samples.

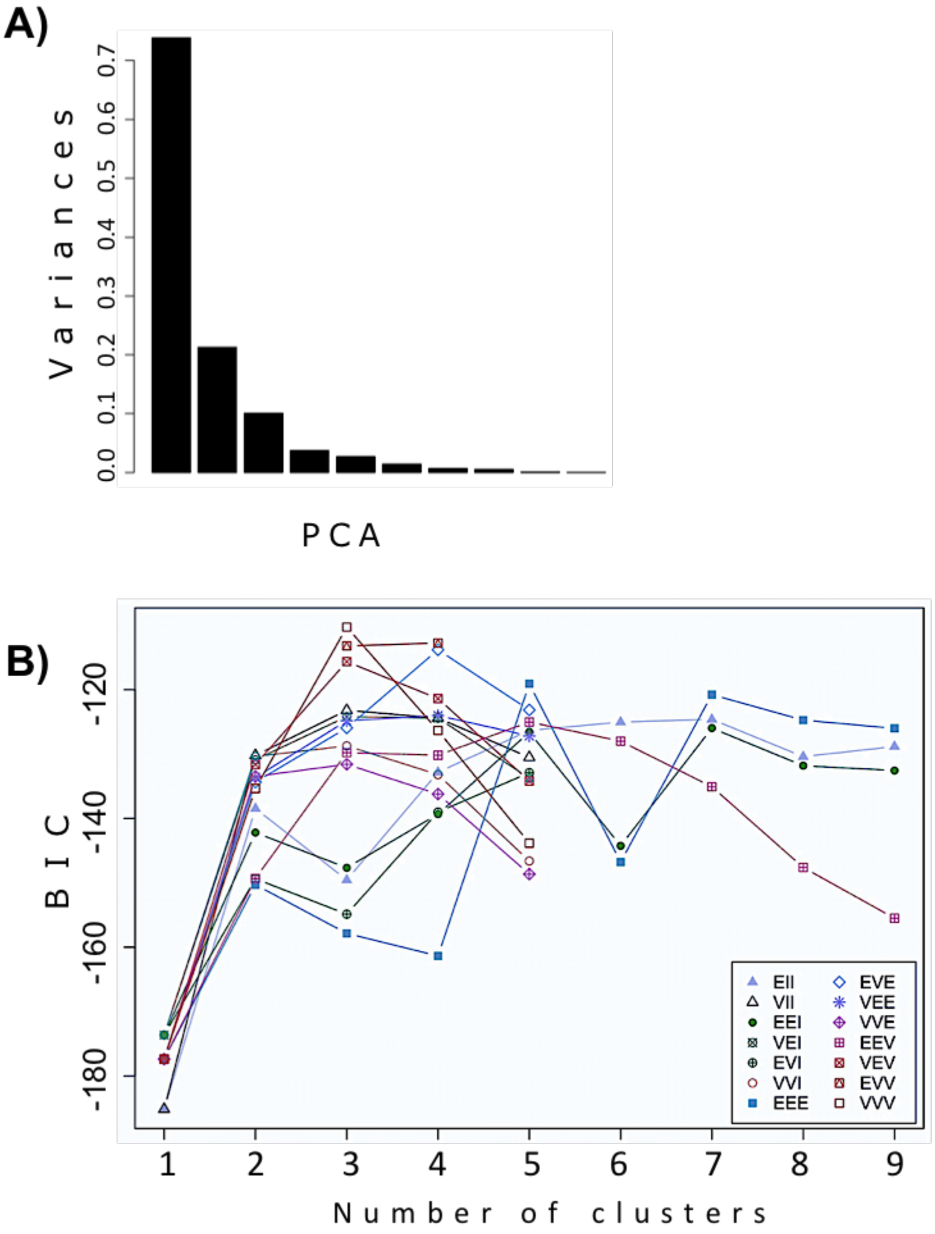

Supplementary Figure S5. Identification of components and protein profiles.

Principal component analysis (PCA), clustering and classification. The $\log_2$ ratios of cytokine intensity values from cells stimulated with [TCR-CD43], [TCR-CD28], [CD43+TCR], [CD28+TCR], [CD43-TCR], and [CD28-TCR] (Figure 2B) were used to obtain the Spearman correlation coefficient between the 42 cytokines. This correlation matrix was the input for PCA. **A)** PCA variance graphed as a scree plot revealed 83% of variance explained by the first two components. **B)** Mclust was used to perform clustering and classification of the first two components of the PCA. The Bayesian Information Coefficient (BIC) score (-110.2721) indicated the unconstrained Gaussian mixture model VVV of three components or clusters as the best-fitting model.

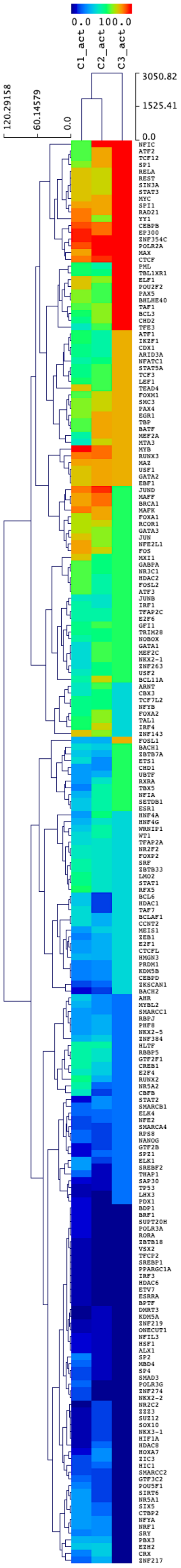
0.0
100.0
C1_act
C2_act
C3_act
3050.82
1525.41
0.0
120.29158
60.14579
0.0
NFIC
ATF2
TCF12
SP1
RELA
REST
SIN3A
STAT3
MYC
SPI1
RAD21
YY1
CEBPB
EP300
ZNF354C
POLR2A
MAX
CTCF
PML
TBL1XR1
ELF1
POU2F2
PAX5
BHLHE40
TAF1
BCL3
CHD2
TFE3
ATF1
IKZF1
CDX1
ARID3A
NFATC1
STAT5A
TCF3
LEF1
TEAD4
FOXM1
SMC3
PAX4
EGR1
TBP
BATF
MEF2A
MTA3
MYB
RUNX3
MAZ
USF1
GATA2
EBF1
JUND
MAFF
BRCA1
MAFK
FOXA1
RCOR1
GATA3
JUN
NFE2L1
FOS
MXI1
GABPA
NR3C1
HDAC2
FOSL2
ATF3
JUNB
IRF1
TFAP2C
E2F6
GFI1
TRIM28
NOBOX
GATA1
MEF2C
NKX2-1
ZNF263
USF2
BCL11A
ARNT
CBX3
TCF7L2
NFYB
FOXA2
TAL1
IRF4
ZNF143
FOSL1
BACH1
ZBTB7A
ETS1
CHD1
UBTF
RXRA
TBX5
NFIA
SETDB1
ESR1
HNF4A
HNF4G
WRNIP1
WT1
TFAP2A
NR2F2
FOXP2
SRF
ZBTB33
LMO2
STAT1
RFX5
BCL6
HDAC1
TAF7
BCLAF1
CCNT2
MEIS1
ZEB1
E2F1
CTCFL
HMGN3
PRDM1
KDM5B
CEBPD
ZKSCAN1
BACH2
AHR
MYBL2
SMARCC1
RBPJ
PHF8
NKX2-5
ZNF384
HLTF
RBBP5
GTF2F1
CREB1
E2F4
RUNX2
NR5A2
CBFB
STAT2
SMARCB1
ELK4
NFE2
SMARCA4
RPS8
NANOG
GTF2B
SPZ1
ELK1
SREBF2
THAP1
SAP30
TP53
LHX3
PDX1
BDP1
BRF1
SUPT20H
POLR3A
RORA
ZBTB18
VSX2
TFCP2
SREBP1
PPARGC1A
IRF3
HDAC6
ETV7
ESRRA
BPTF
DMRT3
KDM5A
ZNF219
ONECUT1
NFIL3
HSF1
ALX1
SP2
MBD4
SP4
SMAD3
POLR3G
ZNF274
NKX2-2
NR2C2
ZZZ3
SUZ12
SOX10
NKX3-1
HIF1A
HDAC8
HOXA7
ZIC3
HIC1
SMARCC2
GTF3C2
POU5F1
SIRT6
NR5A1
SIX5
CTBP2
NFYA
NRF1
SRY
PBX3
EZH2
CRX
ZNF217

Supplementary Figure S6. TF prediction percentages throughout the profiles.

Starting from the matrix of presence and absence of TFs predicted via ENCODE+TRANSFAC, protein-coding genes were ordered based on the profiles defined in C1, C2, and C3 (**Supplementary data Table S4: MatrixTFs & mRNAseqRoadmap**). The frequency of usage of each TF through each profile was calculated to construct a matrix of prediction percentages (**Supplementary data Table S4: TFspercentages**), and the resultant heat map (rainbow scale: blue 0%, red 100%) was ordered from the highest to lowest percentage (**Supplementary data Table S4: MatrixTFsordered percentages**). The hierarchical clustering conditions included the average linkage algorithm and the Manhattan correlation metric.
